# Niosome-Assisted Delivery of DNA Fluorescent Probe with Optimized Strand Displacement for Intracellular MicroRNA21 Imaging

**DOI:** 10.3390/bios12080557

**Published:** 2022-07-24

**Authors:** Zongwei Zhu, Hongqian Zhang, Xiaoxue Dong, Meng Lin, Chuanxu Yang

**Affiliations:** Key Laboratory of Colloid and Interface Chemistry of the Ministry of Education, School of Chemistry and Chemical Engineering, Shandong University, Jinan 250100, China; zhuzongwei@mail.sdu.edu.cn (Z.Z.); hongqianz@mail.sdu.edu.cn (H.Z.); dongxiaoxue@mail.sdu.edu.cn (X.D.)

**Keywords:** miRNA sensing, niosome, fluorescent probe, biosensor, strand displacement reaction

## Abstract

MicroRNAs play a vital role in cancer development and are considered as potential biomarkers for early prognostic assessment. Here, we propose a novel biosensing system to achieve fluorescence imaging of miRNA21 (miR21) in cancer cells. This system consists of two components: an optimized “off-on” double-stranded DNA (dsDNA) fluorescent for miR21 sensing by efficient strand-displacement reaction and a potent carrier vesicle, termed niosome (SPN), to facilitate the efficient intracellular delivery of the dsDNA probe. A series of dsDNA probes based on fluorescence energy resonance transfer (FRET) was assembled to target miR21. By optimizing the appropriate length of the reporter strand in the dsDNA probe, high accuracy and sensitivity for miR21 recognition are ensured. To overcome the cellular barrier, we synthesized SPN with the main components of a nonionic surfactant Span 80 and a cationic lipid DOTAP, which could efficiently load dsDNA probes via electrostatic interactions and potently deliver the dsDNA probes into cells with good biosafety. The SPN/dsDNA achieved efficient miR21 fluorescent imaging in living cells, and could discriminate cancer cells (MCF-7) from normal cells (L-02). Therefore, the proposed SPN/dsDNA system provides a powerful tool for intracellular miRNA biosensing, which holds great promise for early cancer diagnosis.

## 1. Introduction

MicroRNAs (miRNA) are endogenous, short (about 19–24 nucleotides), single-stranded, non-coding RNAs that can bind to target mRNAs and regulate gene expression at the post-transcriptional level [1]. An increasing number of studies have demonstrated that the deregulation of miRNA expression is closely associated with the initiation and progression of a variety of diseases, including tumorigenesis, and miRNAs are regarded as potential cancer biomarkers [2,3]. Therefore, the monitoring and sensing of the miRNAs in living cells is essential for cancer diagnosis, as well as understanding their tumor-related biological functions [4,5,6,7,8]. Among all miRNAs, miR-21-5p (miR21) is highly expressed and exhibits a strong correlation with various types of cancer and has been considered as a potential clinical diagnostic biomarker [9,10,11,12]. For example, miR21 is reported to be significantly overexpressed in a variety of cancer cells or tumors, including hepatocellular carcinoma, glioblastoma, lung cancer, ovarian cancer and B-cell lymphoma, which is associated with high proliferation, invasion, oncogenesis and metastatic potential [13,14]. In addition, miR21 is found consistently upregulated in both animal models and clinical patient samples [13,15,16]. Notably, miR21 has also been reported to be a predictive biomarker for at least 29 diseases. It is not an ideal fluid-based, specific biomarker, as the best miRNA biomarkers should indicate specific injury or perturbation [17,18]; however, the intracellular sensing of miR21 could still provide some valuable information during prognosis or functional studies.

Fluorescent probes, which convert diverse target activities into fluorescence readouts, are widely used for biosensing applications due to their high sensitivity, high resolution and fast responses [19,20,21]. To construct fluorescent probes for biosensing of miRNA, DNA oligonucleotides, as a powerful programmable material, can be designed and assembled with a special spatial structure with specific recognition ability, which renders DNA probes a powerful tool for target miRNA detection [22,23]. In addition, functional DNA is easily labeled with fluorescent molecules or materials, which is suitable for quantification and localization during the sensing process [24]. Several DNA-based fluorescence miRNA imaging techniques have been developed, including fluorescence in situ hybridization (FISH) assay and hybridization chain reaction (HCR) assay [25,26,27,28,29]; however, these procedures usually require cell pre-fixation to increase permeability or the introduction of exogenous enzymes, making intracellular miRNA imaging in living cells difficult. Therefore, the development of selective and efficient intracellular imaging of miRNA fluorescent probes remains a great challenge [30].

The cellular barriers, including cell membrane and endosome entrapment, significantly impede DNA probes’ internalization and cytosol transportation [31], which presents severe challenges for intracellular miRNA sensing. Therefore, the development of a proper carrier system to facilitate efficient DNA probes’ intracellular delivery is crucial for realizing miRNA biosensing in cells. Cationic liposomes are widely used for DNA delivery and cell transfection due to their positively charged properties; however, their significant cytotoxicity limits their clinical applications [32,33,34]. Nonionic surfactant vesicles, termed “niosomes”, have recently been shown to be promising nucleic-acid-based drug delivery vehicles [35,36]. Compared with traditional liposomes, niosomes have the advantages of higher stability and loading capacity and enhanced endosome-escaping capability [35,36]. In addition, as a major component of niosomes, nonionic surfactants such as sorbitan monooleate (span 80) demonstrate low cytotoxicity and good biocompatibility and are widely used in the pharmaceutical, food and cosmetics industries [37,38,39,40]. Therefore, the application of niosomes for DNA probe delivery to overcome cellular barriers might be promising for intracellular miRNA biosensing; however, this has not yet been investigated.

**Scheme 1:**
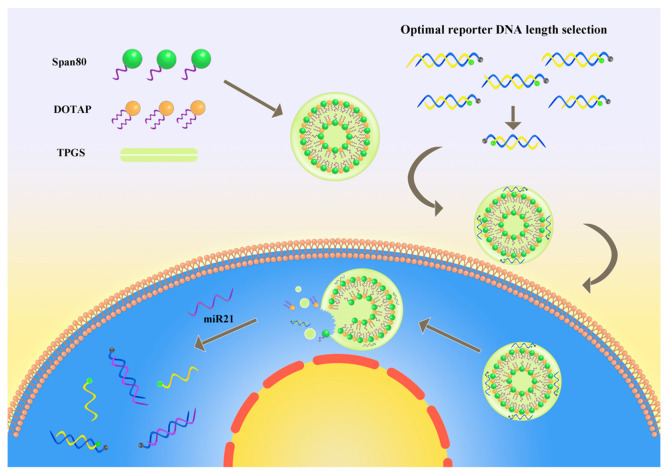
Schematic illustration showing the optimization and assembly process of niosome/dsDNA platform and miR21 biosensing in cancer cells.

Here, we have developed a novel SPN/DNA platform for sensing and imaging miR21 in tumor cells. As illustrated in Scheme 1, FAM-labeled reporter DNA (DNA-FAM) forms double-stranded DNA in the presence of BHQ1-labeled capture DNA (DNA-BHQ1) to turn off fluorescence, due to fluorescence resonance energy transfer (FRET). The lengths of reporter DNA are optimized to achieve efficient strand displacement reactions with miR21 to restore the fluorescence signal, which exhibited high sensitivity and accuracy for in vitro miR21 sensing. In addition, to overcome the cellular barrier, niosome (SPN) was synthesized as a dsDNA carrier, which consists of PEGylated α-tocopherol (TPGS), nonionic surfactant Span80 and cationic lipid DOTAP. The SPN/dsDNA complexes achieved efficient miR21 sensing in living cells and could discriminate normal cells (L-O2) from cancer cells (MCF-7). Compared with other intracellular fluorescence detection platforms, the SPN/dsDNA developed here is particularly attractive due to its easy preparation and efficient transportation of DNA probes across cellular barriers to realize intracellular miRNA imaging.

## 2. Materials and Methods

### 2.1. Materials

D-α-Tocopheryl polyethylene glycol-1000 succinate (TPGS) was supplied by Sigma-Aldrich. 1,2-Dioleoyl-3-trimethylammonium-propane (DOTAP) was supplied by Avanti Polar Lipids (Alabaster, AL, USA). Sorbitan monooleate (Span 80) was supplied by Macklin Biochemical Co., Ltd. (Shanghai, China). Dulbecco’s modified eagle medium (DMEM), Roswell Park Memorial Institute 1640 (RPMI-1640) and fetal bovine serum (FBS) were obtained from Gibco (Grand Island, NY, USA). All the DNA oligonucleotides (Table 1) were synthesized by Sangon Biotech (Shanghai, China).

### 2.2. Preparation of Double-Stranded DNA Probe

The double-stranded DNA (dsDNA) probes used for detection were formed by the annealing of partially complementary single-stranded DNAs (ssDNA) labeled with fluorophore FAM or quencher BHQ-1, respectively, which are suitable for energy resonance transfer. First, the FAM-labeled ssDNA (DNA-FAM) and the BHQ1-labeled ssDNA (DNA-BHQ1) were mixed in the DNA annealing buffer solution at a concentration of 1:1. Next, the solution was rapidly heated to 95 °C and held for 2 min, then slowly cooled to room temperature, during which dsDNA was formed by annealing. Finally, the dsDNA was stored at 4 °C before use.

### 2.3. Preparation and Characterization of Niosome System

Empty niosome (SPN) was synthesized by nanoprecipitation method according to previous work [40]. Specifically, sorbitan monooleate (Span 80), 1,2-dioleoyl-3-trimethylammonium-propane (DOTAP) and D-α-Tocopheryl polyethylene glycol-1000 succinate (TPGS) were completely dissolved in anhydrous ethanol. These components were premixed in a molar ratio of 50:45:5 (DOTAP/Span 80/TPGS). The mixture was then dropwise injected into deionized water in a moderate vortex to ensure uniform particle synthesis. Empty SPN was assembled and stored at 4 °C before subsequent use.

To load dsDNA probes, Milli-Q water, DNA probe stock solution (20 μM) and SPN were added sequentially in a volume ratio of 50:15:35. The components were fully dispersed during the dilution and mixing process, and the mixture was stored at room temperature for 15 min before use.

### 2.4. Characterization of SPN and SPN/dsDNA Complexes

The SPN and SPN/DNA system was characterized by dynamic light scattering (DLS) using a Zetasizer Nano ZS (Malvern, UK). Briefly, SPN (1 mg/mL) was incubated with dsDNA at the ratios of 1:5, 1:10, 1:15, 1:20 and 1:25 for 1 h and diluted with Milli-Q water for DLS and TEM test. The size and morphology of SPN was examined by transmission electron microscopy (TEM, FEI Tecnai G2 Spirit, OR, USA).

Encapsulation efficiency of dsDNA by SPN was determined by agarose gel electrophoresis. DNA with the same concentration (5 μM) was incubated with SPN for 1 h at the ratios of 1:2.5, 1:5, 1:10, 1:15 and 1:20 and loaded onto agarose gel (2% agarose). The gel was operated at 120 V for 15 min in the electrophoresis tank with a DC power supply. The gel was stained with SYBR Gold and imaged using the Gel Doc EZ System (Bio-Rad Laboratories Inc., CA, USA).

### 2.5. Selection of Reporter DNA Strand Length

Reporter DNA (DNA-FAM) of different lengths and capture DNA (DNA-BHQ1) were used to assemble dsDNA probes. Then, dsDNA (100 nM) was co-incubated with miR21 (100 nM) in buffer (2 mM MgCl_2_, 50 mM NaCl, 10 mM Tris, pH = 7.40) at 37 °C for 1 h in dark. The fluorescence spectra of dsDNA were then measured at the excitation wavelength of 480 nm by fluorescence spectrometer (F-7100, Hitachi, Japan). The emission spectra were collected from 490 nm to 650 nm to obtain complete peak spectra.

### 2.6. Fluorescence Assay of miR21

Different concentrations of miR21 were added to dsDNA (100 nM) containing the 16-base reporter DNA. The fluorescence spectra of FAM were recorded after incubation at 37 °C for 1 h in dark, and the fluorescence intensity values were obtained from 520 nm. For probe specificity analysis, mismatched miRNAs or other miRNAs were introduced during the fluorescence assay, and the fluorescence intensity was recorded and compared to that of miR21. The fluorescence spectra were collected by fluorescence spectrometer (F-7100, Hitachi, Japan).

### 2.7. Cell Culture

Human breast cancer cell line MCF-7 and hepatocyte cell line L-O2 were utilized for fluorescence imaging experiments. The MCF-7 cells were maintained in Dulbecco’s modified Eagle’s medium (DMEM) with 10% fetal bovine serum (FBS) and 1% penicillin/streptomycin (P/S) at 37 °C, 5% CO_2_ and 100% humidity. The L-O2 cells were cultured in Roswell Park Memorial Institute 1640 (RPMI-1640) with 10% FBS and 1% P/S under the same conditions.

### 2.8. miRNA Quantification by RT-qPCR

The expression levels of miR21 were quantified in both MCF-7 and L-O2 cells by RT-qPCR. First, total RNA from cells was extracted using Takara RNAiso Plus according to the manufacturer protocol. Next, the concentrations of miR21 in total RNA samples were determined using Hairpin-it RT-PCR kit assay (GenePharma, Shanghai, China) according to the manufacturer manual, and intracellular snRNA U6 was used as an internal reference. RT-qPCR was performed on a LightCycler 480 II Real-time PCR system (Roche, Switzerland).

### 2.9. In Situ Fluorescence Imaging of miR21

For confocal scanning microscopy imaging, cells were seeded at a concentration of 1 × 10^5^ cells/mL in glass bottom confocal dishes at 37 °C, 5% CO_2_ and maintained overnight. Then, the medium was replaced and SPN-DNA solution was added to the final probe concentration of 100 nM and cultured for 4 h. Next, the treated cells were fixed with 4% paraformaldehyde solution, and Hoechst 33342 (2 μg/mL) was used to stain the nucleus. Then, the cells were imaged using a confocal laser microscope (TCS SP8, Leica, Germany).

To analyze the intracellular fluorescence heterogeneity, the green fluorescence of FAM signals from confocal fluorescence images was subjected to intensity quantification statistics by ImageJ software. Fluorescence quantification was performed on a sufficient number of cells (>100) and the fluorescence intensities were obtained by subtracting the background signal (average signal intensity in untreated cells). The cell fluorescence intensity histogram was plotted using OriginPro 2022b (https://www.originlab.com/, accessed on 23 June 2022).

### 2.10. Cell Viability

Cell viability was evaluated by using Cell Counting Kit-8 (CCK-8) according to the manufacturer’s protocol. Cell suspensions (100 μL, 1 × 10^4^ cells/well) were added to a 96-well plate and incubated for 24 h. DNA, SPN and SPN-DNA samples were added to the wells for 4 h at predetermined concentration. Then, the medium was changed and the cells were cultured for 24 h before CCK-8 solution (10 μL) was added to each well. After 1 h, the absorbance of the wells of the 96-well plate was measured at 450 nm by a microplate (Infinite F50, Tecan, Switzerland) and used to calculate the cell viability.

### 2.11. Statistical Analysis

Data are presented as mean ± S.D. Statistical analysis was performed using an unpaired Student’s *t*-test, and statistical difference was defined as * *p* < 0.05, ** *p* < 0.01, *** *p* < 0.001.

## 3. Results and Discussion

### 3.1. Optimization of Double-Stranded DNA Probe for miR21 Sensing

The dsDNA probes for targeting miR21 were assembled by annealing the FAM-labeled reporter ssDNA (DNA-FAM) and BHQ1-labeled capture ssDNA (DNA-BHQ1). FAM and BHQ1 were effective fluorescent resonance energy transfer (FRET) donors and receptors, and the FRET efficiency was mainly affected by the distance between the energy donor and acceptor. For miRNA sensing, the competitive displacement of the reporter strand (DNA-FAM) by miRNA could trigger the FAM signal recovery. As illustrated in Scheme 1, the sequence of the capture DNA (DNA-BHQ1) was designed to be completely complementary with miR21, which was annealed to the reporter DNA (DNA-FAM) to assemble the dsDNA probe. For the design of the dsDNA probe, the FAM molecule was modified at the 5′ end of the reporter DNA and the BHQ1 was modified at the 3′ end of the capture DNA to ensure the proximity of the donor-acceptor and the FRET efficiency. To further optimize the sensitivity of the dsDNA probe, the reporter DNA (DNA-FAM), with different DNA lengths ranging from 22 to 14 nt and with gradually reduced bases at the 3′ end, was annealed to the capture strand (DNA-BHQ1), which might affect the probe stability and the desired miR21-DNA displacement efficiency.

**Figure 1:**
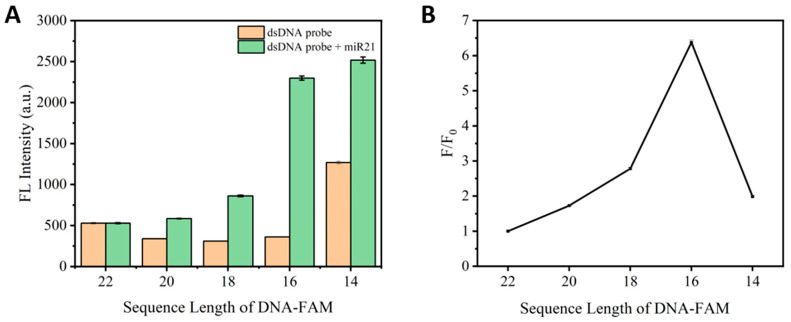
Length optimization of reporter DNA strand (DNA-FAM) for efficient strand displacement reaction towards miR21. (**A**) Effect of different reporter probe lengths for the fluorescence intensity of dsDNA probe (100 nM) or dsDNA probe (100 nM) supplemented with miR21 (100 nM) and (**B**) the ratio of recovered fluorescence after introducing the target miR21.

After the assembly of the dsDNA probes, the fluorescence spectra of each probe before and after introduction of miR21 was determined, and the fluorescence intensity at 520 nm was measured for the calculation of fluorescence recovery efficiency. The fluorescence intensity of the annealed dsDNA probes was effectively quenched due to the FRET effect (Figure 1A and Figure S1), except for that of the probe with the 14 nt reporter DNA, which might be due to its low annealing efficiency to the capture strand. Then, the introduction of miR21 restored the fluorescence signal, indicating that target recognition and strand displacement happens. More importantly, the restored fluorescence signal gradually increased with the shortening of the length of the DNA-FAM strand due to the enhanced strand displacement by miR21, which led to the efficient separation of capture and reporter DNA. The recovery efficiency of fluorescence intensity for each probe was further calculated, which first increased and then decreased with the shortening of the length of the reporter DNA and reached its peak (~6.4 fold) at the 16 nt reporter DNA (Figure 1B), indicating the best signal-to-ratio of the probe.

The fluorescence spectrum of the optimized probe with 16 nt reporter DNA is shown in detail in Figure S1. The fluorescence of the reporter DNA (DNA-FAM) was effectively excited at the 480 nm excitation wavelength, and the introduction of capture DNA (DNA-BHQ1) effectively quenched the fluorescence. After introducing miR21, the quenched fluorescence signal recovered to 97% of the original reporter DNA-FAM fluorescence intensity, which proved that efficient competitive strand displacement occurred and that the FRET effect was relieved. In addition, the stability of the optimized dsDNA probe was also evaluated. The target recognition and fluorescent signal recovery efficiency was kept almost unchanged during 14 days of storage at 4 °C (Figure S2), indicating the good stability of the optimized dsDNA probe. Based on the results above, the 16 nt reporter DNA-FAM strand represented an optimized length that balanced dsDNA probe stability and target sensitivity, and it was chosen to construct the final sensing system.

### 3.2. Analytical Performance of the dsDNA Probe for In Vitro miR21 Sensing

**Figure 2:**
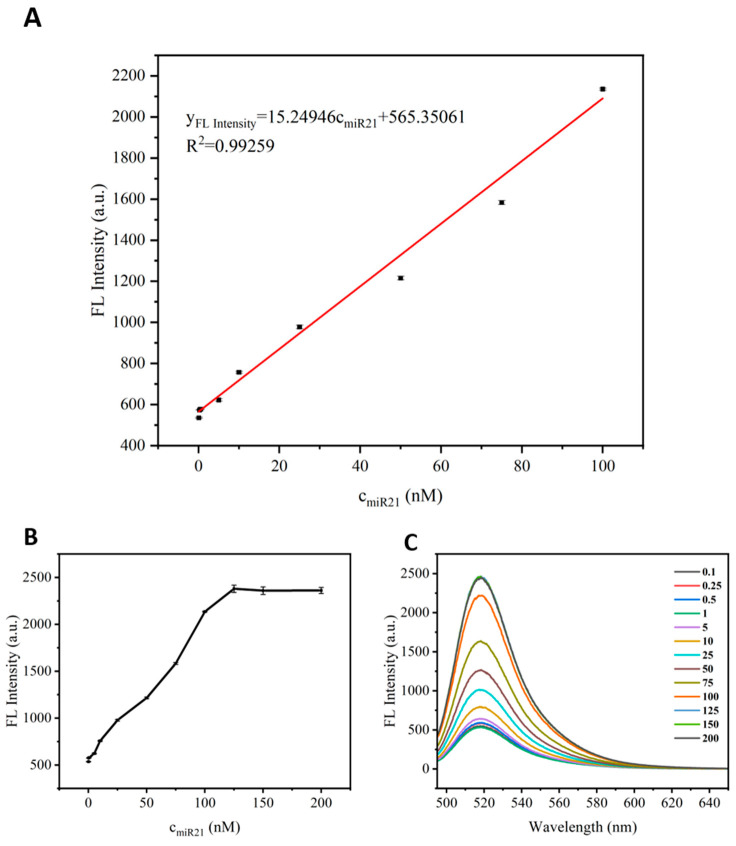
In vitro detection miR21 by the sequence optimized dsDNA probe. (**A**) The linear relationship between FL intensity and c_miR21_ ranging from 0.1 to 100 nM at a dsDNA probe concentration of 100 nM, (**B**) fluorescence intensity of DNA probes with the miR21 at indicated concentrations and (**C**) the corresponding fluorescence spectra.

The analytical performance of the competitive DNA probe for the detection of miR21 was evaluated using in vitro sensing experiments. MiR21 with pre-determined concentrations was added to the optimized dsDNA probe and incubated at 37 °C for 1 h in dark, and then the fluorescence spectra were recorded at the excitation wavelength of 480 nm. The working curve was determined by the fluorescence intensity of each sample at the maximum emission wavelength of 520 nm. According to the working curve (Figure 2A), the concentration of miR21 showed a linear relationship with the fluorescence intensity in the range of 0.1 nM to 100 nM at the probe concentration of 100 nM, and the limit of detection (LOD) was 0.068 nM according to the blank response. The linear regression equation (c, nM) was y_FL Intensity_ = 15.24946c_miR__21_ + 565.35061 (correlation coefficient, R^2^ = 0.99259). After the concentration of miR21 reached 100 nm, the increase in fluorescence intensity was no longer proportional to the miR21 concentration, and it reached a plateau after 125 nm due to the saturation of the target against the probes (Figure 2B). The sensing range and detection limit of the dsDNA probe were comparable to reported systems [41,42,43,44,45]. In addition, the fluorescence spectra of the sensing system with different concentrations of miR21 demonstrated concentration-dependent fluorescence recovery and consistency in the emission curve (Figure 2C). These results confirmed that the optimized dsDNA probe could sensitively monitor the miR21 level in vitro.

### 3.3. Selectivity and Specificity of the dsDNA Probe

**Figure 3:**
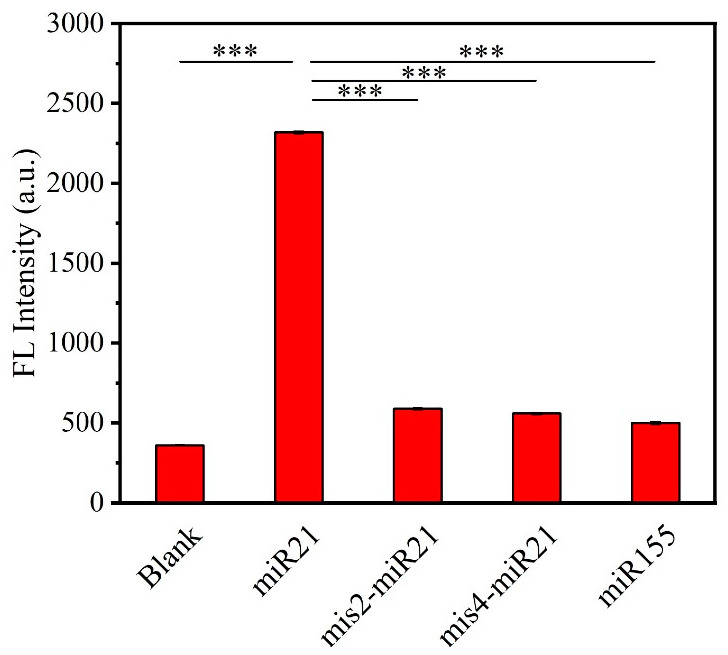
Selectivity and specificity analysis of dsDNA probe. The fluorescence intensity of dsDNA probe (100 nM) supplemented with buffer (Blank), miR21, two-base-mismatched (mis-2-miR21), four-base-mismatched (mis-4-miR21) and miR155 RNAs. Results represent mean ± SD (n = 3). Statistical difference, *** *p* < 0.001.

The selectivity and specificity of the probe is another critical issue to be considered for successful miRNA sensing due to the complexity of the intracellular environment and RNA species. To verify the specificity of the probe, we designed several mismatched or non-relevant miRNA targets to challenge the dsDNA probe. The miR21 or nontarget RNAs were incubated with the dsDNA probe at the concentration of 100 nM and kept in the dark at 37 °C for 1 h before being analyzed by a fluorometer. As shown in Figure 3, miR21 significantly recovered the fluorescence intensity. In contrast, other nontarget RNAs, including two-base-mismatched (mis-2-miR21), four-base-mismatched (mis-4-miR21) and miR155 RNAs, exhibited very low fluorescence intensity, close to that of the bare probe solution. This result demonstrated the good selectivity and specificity of the dsDNA probe towards miR21, which is crucial for further biosensing applications.

### 3.4. Synthesis and Characterization of Niosome/dsDNA Platform

**Figure 4:**
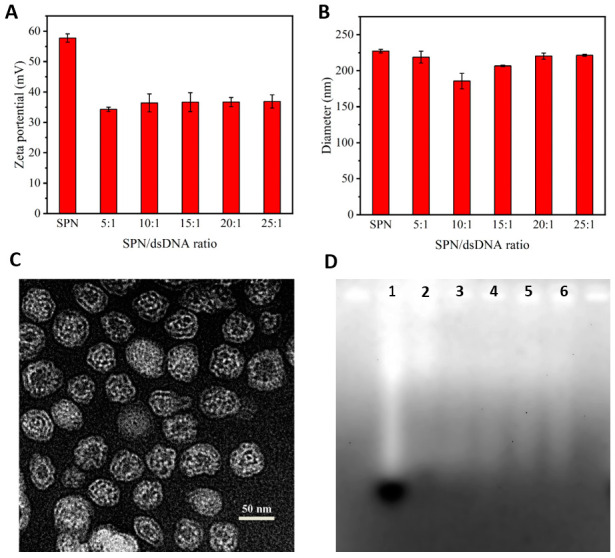
Characterization of SPN. (**A**) Hydrodynamic diameter and (**B**) zeta potential of SPN and SPN/DNA complexes measured by DLS. Data represent mean ± SD (n = 3). (**C**) TEM images of SPN. Scale bar, 50 nm. (**D**) Gel retardation assay for the detection of the loading of DNA probe to SPN. Lane 1, free dsDNA as control; lanes 2−6, SPN/DNA complexes prepared at weight ratios (SPN/DNA, *w*/*w*) of 2.5, 5, 10, 15 and 20, respectively.

Cellular barriers, including cell membrane and endosome entrapment, significantly hinder dsDNA probes’ internalization and cytosol transportation, which presents severe challenges for intracellular miR21 sensing. To overcome these barriers, we synthesized a nonionic surfactant vesicle (niosome) for intracellular dsDNA probe delivery. As illustrated in Scheme 1, niosome (SPN) was formulated with nonionic surfactant Span 80, cationic lipid DOTAP and a PEGylated vitamin E (TPGS) via nanoprecipitation method. During the process of solvent exchange, the amphiphilic components self-assembled into liposome-like vesicles from the hydrophobic interactions. To verify the successful assembly of niosomes, dynamic light scattering (DLS) was used for size characterization. The obtained SPN exhibited a size of 227 nm and a zeta potential of +57.7 mV (Figure 4A,B). The DLS characterization of the hydrodynamic diameter indicated the formation of nanosized SPN. TEM images showed that SPN formed a spherical, multilayer structure similar to multilamellar liposomes, and the particle size was relatively uniform (Figure 4C). In addition, the positive surface charge of SPN is due to the presence of cationic lipids DOTAP, which might facilitate the electrostatic interaction with the dsDNA probe.

Next, we investigated the dsDNA probe loading capability by SPN. SPN/dsDNA complexes formulated at different ratios of 5:1, 10:1, 15:1, 20:1 and 25:1 (SPN/DNA, *w*/*w*) were characterized by DLS and gel electrophoresis. Negatively charged dsDNA and positively charged SPN were complexed by electrostatic interaction, which might influence the particle size and zeta potential. From the DLS measurement, the hydrodynamic diameter of the SPN/dsDNA showed a trend of first decreasing and then increasing, reaching the lowest diameter of 186 nm at the ratio of 10:1 (Figure 4A). The zeta potential of SPN decreased significantly after the introduction of dsDNA (Figure 4B), which might be due to the binding and charge neutralization by dsDNA. To intuitively evaluate the dsDNA loading efficiency by SPN, agarose gel electrophoresis was conducted for separation of the complexed dsDNA and free dsDNA probe. As shown in Figure 4D, compared with free dsDNA, SPN/dsDNA exhibited significantly lower fluorescence (black) intensity at the position of free dsDNA, and no free dsDNA was observed when the ratio reached 10, which proved that dsDNA was effectively complexed by SPN. SPN/dsDNA formulated at ratio 10 was further utilized for cell studies.

### 3.5. Intracellular miR21 Fluorescence Imaging

**Figure 5:**
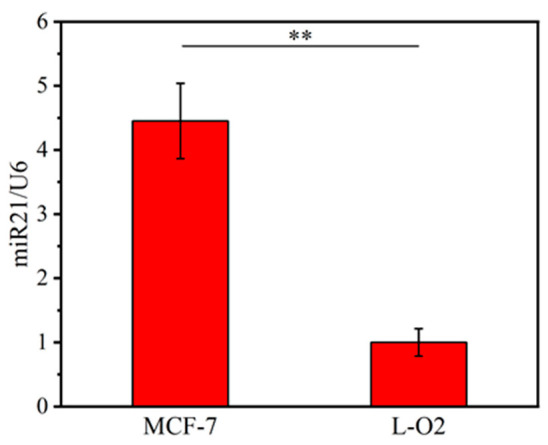
Expression level of miR21 in MCF7 and L-O2 cells. RNA isolated from cells was analyzed by RT-qPCR, and the average expression level of miR21 was normalized to U6 snRNA. Results represent mean ± SD (n = 3). Statistical difference, ** *p* < 0.01.

To verify the imaging effect of the SPN/DNA system, we selected two cell lines, MCF-7 and L-O2. MCF-7, derived from breast cancer, is a widely used cancer cell line, which is reported to have a high expression of miR21 and has been used as a target cell line in many studies. The L-O2 cell line is derived from normal human hepatocytes and is often used as a negative control [4,46,47,48]. First, we quantified the miR21 expression level in both cells using RT-qPCR with intracellular snRNA U6 as an internal reference. RT-qPCR results showed that the expression level of miR21 in tumor cell MCF-7 was 4.5 times greater than in normal cell L-O2 (Figure 5), proving that the two cell lines are suitable for the miR21 sensing experiment.

**Figure 6:**
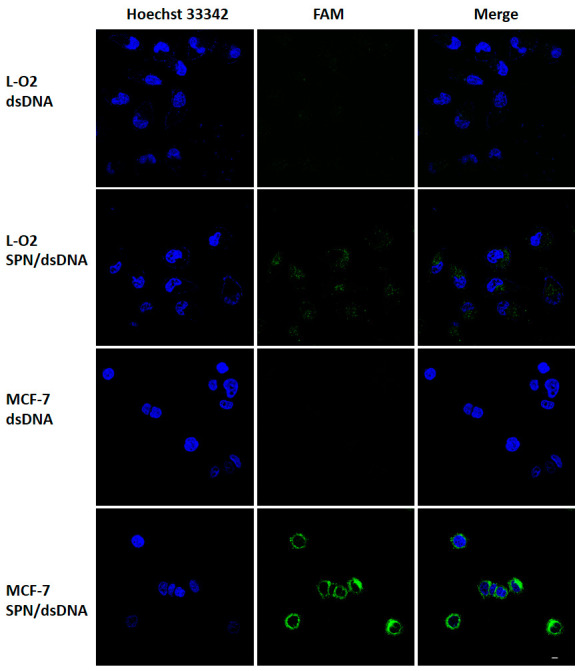
Intracellular imaging of miR21 by SPN/dsDNA probe. Confocal images of MCF-7 and L-O2 after incubation with SPN/dsDNA for 4 h. Cell nucleus stained with Hoechst 33,342 (blue; excitation, 405 nm; emission, 420–667 nm) and reporter DNA labeled with FAM (green; excitation, 488 nm; emission, 504–697 nm). Scale bar, 10 μm.

For intracellular detection of miR21, SPN/dsDNA was freshly prepared at a ratio of 10 and supplemented to cells at a final dsDNA concentration of 50 nM. MCF-7 or L-O2 cells were incubated with SPN/dsDNA for 4 h, and miR21 bioimaging was then performed using a confocal microscope. Fluorescence signals of the blue and green channels were acquired, demonstrating the labeled nuclei in blue and FAM-labeled DNA in green. As shown in Figure 6, no obvious FAM fluorescent signal was detected in cells incubated only with dsDNA, neither in normal nor in tumor cells, which is due to the low intracellular uptake of the dsDNA probe, and no intracellular miR21 could be sensed. In contrast, MCF-7 cells treated with SPN/dsDNA exhibited strong FAM fluorescence signals inside the cells, while in comparison, only slight FAM signals were observed in L-O2 cells. The results from SPN/dsDNA-mediated intracellular miR21 fluorescence imaging are consistent with the different miR21 expression level from RT-qPCR analysis, which could be mainly attributed to the combined effects of efficient intracellular transportation of dsDNA mediated by SPN and the accuracy and high specificity of the optimized dsDNA probe towards miR21.

A clear advantage of intracellular fluorescent imaging over classical bulk analysis by qPCR is the ability to directly observe cellular heterogeneity. Therefore, we further quantified the fluorescent signals in individual cells from the fluorescence microscopy with a sufficient number of cells (>100), and the obtained histogram showed the fluorescent signals in the cells distributed relatively narrowly with one peak (Figure S3). This indicates the relative heterogeneity of the expression levels of miR21 in individual cells. In addition, by quantification of the mean fluorescence intensity from the histogram (Figure S3), the average fluorescence of MCF-7 cells was about four times that of L-O2 cells (Figure S4), which is comparable to that measured by RT-qPCR. In addition, a cell viability experiment was conducted, and no obvious cell toxicity for either MCF-7 or L-O2 cells was observed after SPN/dsDNA treatment, indicating the good biosafety of the SPN/dsDNA system (Figure S5).

From the results above, the SPN/dsDNA system shows great potential for intracellular miR21 imaging to discriminate normal cells from cancer cells. Moreover, by simply changing the corresponding dsDNA probe sequences, the SPN/dsDNA system can easily be extended to detect various intracellular miRNAs for functional studies or diagnosis purposes.

## 4. Conclusions

In this study, we have proposed a novel biosensing system to realize the fluorescence imaging of miR21 in tumor cells. This system consists of two major parts: an “off-on” dsDNA probe for miR21 sensing by efficient strand-displacement reaction and niosome vesicles (SPN) to facilitate efficient intracellular delivery of the dsDNA probe. The dsDNA probe with optimized detection strand length achieved efficient strand displacement reactions with miR21 and resulted in highly sensitive and specific miR21 quantification in vitro. The SPN could potently deliver dsDNA probes into cells with good biosafety. When applied to cells, SPN/dsDNA achieved efficient miR21 sensing in living cells, which could discriminate normal cells from cancer cells. The developed SPN/dsDNA system represents a powerful tool for the detection of various intracellular miRNAs by simply changing the dsDNA sequences, which holds great potential for both biomedical research and clinical disease diagnosis.

## Figures and Tables

**Table 1:** Sequences of DNA or RNA used in the experiments.

Name	Sequence 5′-3′
Capture DNA	TCAACATCAGTCTGATAAGCTA-BHQ1
Reporter DNA 22	FAM-TAGCTTATCAGACTGATGTTGA
Reporter DNA 20	FAM-TAGCTTATCAGACTGATGTT
Reporter DNA 18	FAM-TAGCTTATCAGACTGATG
Reporter DNA 16	FAM-TAGCTTATCAGACTGA
Reporter DNA 14	FAM-TAGCTTATCAGACT
miR21	UAG CUU AUC AGA CUG AUG UUG A
mis-2-miR21	UAG CUU AUG AGA GUG AUG UUG A
mis-4-miR21	UAG CUU AUG ACA GUG AUC UUG A
miR155	UUA AUG CUA AUU GUG AUA GGG GU

## Data Availability

Data are contained within the article.

## Supplementary Materials

The following supporting information can be downloaded at: https://www.mdpi.com/article/10.3390/bios12080557/s1, Figure S1: Fluorescence properties of DNA probes; Figure S2: Stability analysis of dsDNA probe; Figure S3: Histogram of the expression levels of miR21 in individual cells; Figure S4: Quantification the relative expression of miR21 level from intracellular imaging; Figure S5: Cytotoxicity analysis of SPN/dsDNA system.
