# Supplementary material for: Niosome-Assisted Delivery of DNA Fluorescent Probe with Optimized Strand Displacement for Intracellular MicroRNA21 Imaging
Source: Biosensors (Basel). 2022 Jul 24;12(8):557. doi: 10.3390/bios12080557 (PMC9331323; doi:10.3390/bios12080557)
Supplement: Supplementary file 1 [file biosensors-12-00557-s001.zip › biosensors-1818965-supplementary.pdf]

biosensors

MDPI

Supporting Information: Niosome-Assisted Delivery of DNA Fluorescent Probe with Optimized Strand Displacement for Intracellular MicroRNA21 Imaging

Zongwei Zhu, Hongqian Zhang, Xiaoxue Dong, Meng Lin * and Chuanxu Yang *

Key Laboratory of Colloid and Interface Chemistry of the Ministry of Education, School of Chemistry and Chemical Engineering, Shandong University, Jinan 250100, China; zhuzongwei@mail.sdu.edu.cn (Z.Z.); hongqianz@mail.sdu.edu.cn (H.Z.); dongxiaoxue@mail.sdu.edu.cn (X.D.)

* Correspondence: mlin@sdu.edu.cn (M.L.); chuanxu@sdu.edu.cn (C.Y.)

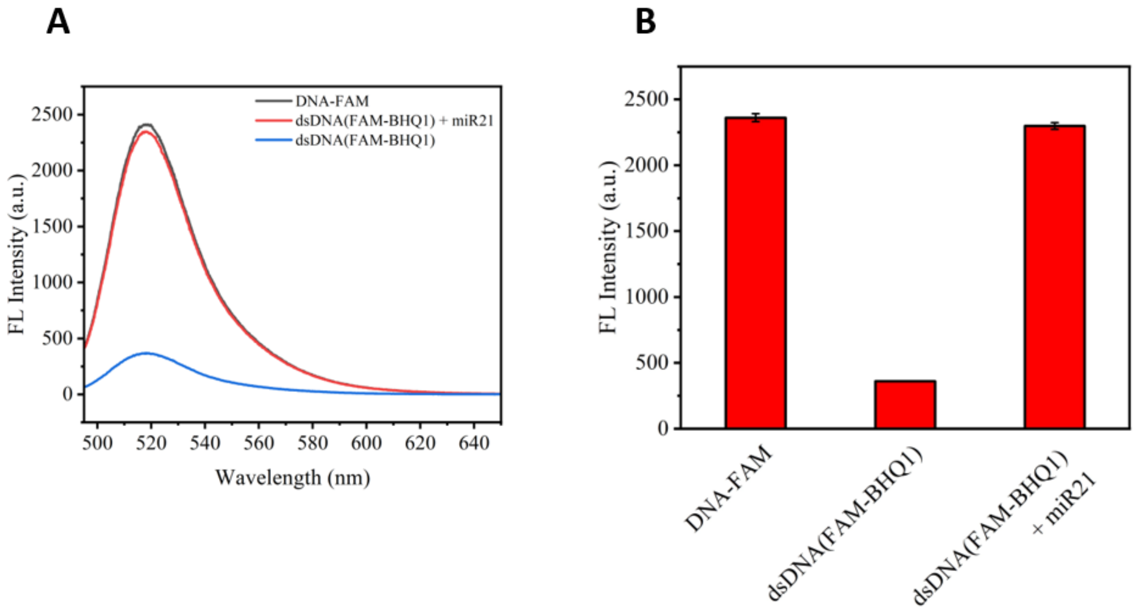

Figure S1. Fluorescence properties of DNA probes. (A) Fluorescence spectra and (B) fluorescence intensity of DNA-FAM (100 nM), dsDNA probe (100 nM), and dsDNA probe (100 nM) supplemented with miR21 (100 nM).

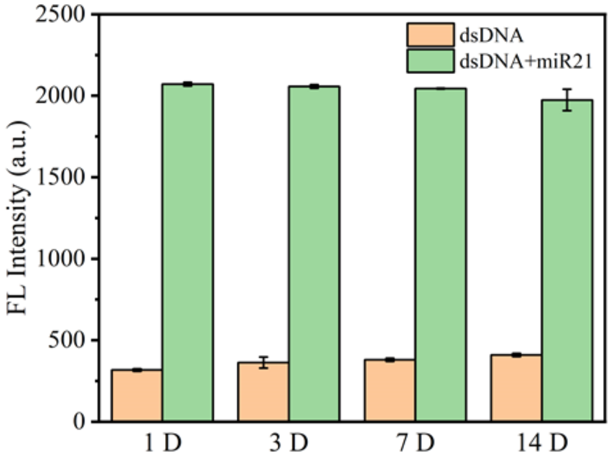

Figure S2. Stability analysis of dsDNA probe. The dsDNA probe was stored at 4°C for indicated period (1D, 1 day; 2D, 2 days; 7D, 7 days; 14D, 14 days) and the target (miR21) recognition capability was evaluated by measuring the fluorescent intensity (emission, 520 nm) before and after introducing of miR21 (100 nM).

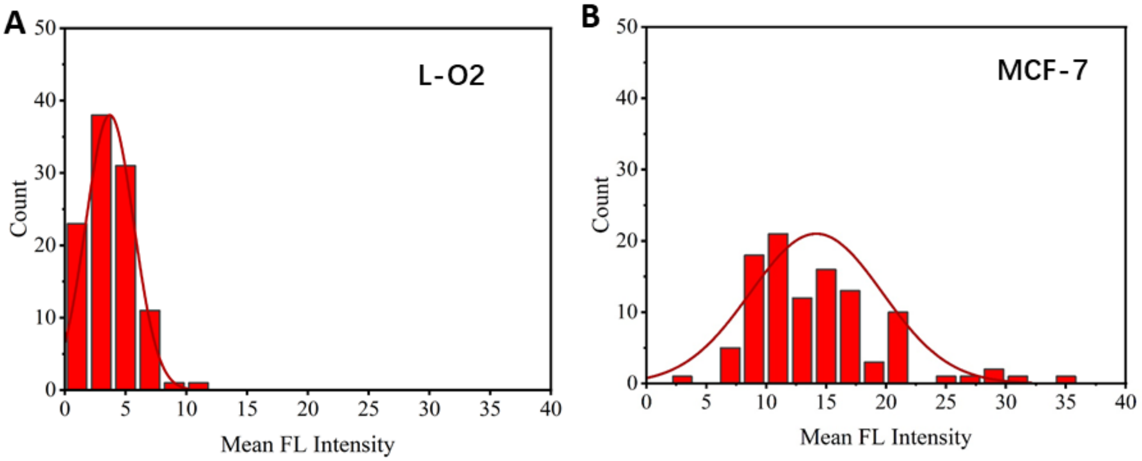

Figure S3. Histogram of the expression levels of miR21 in individual cells. The heterogeneity and distribution of miR21 expression in individual cells was demonstrated by quantifying the fluorescent intensity of sufficient number of cells (>100) from intracellular imaging.

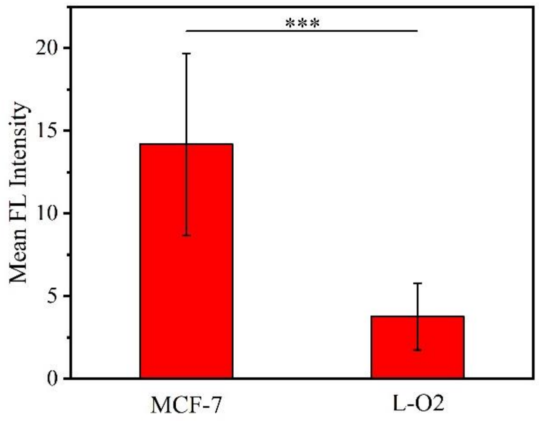

Figure S4. Quantification the relative expression of miR21 level from intracellular imaging. Relative expression of miR21 level was quantified from the mean fluorescent intensity of sufficient number of cells from intracellular imaging. Statistical difference, ***p < 0.001.

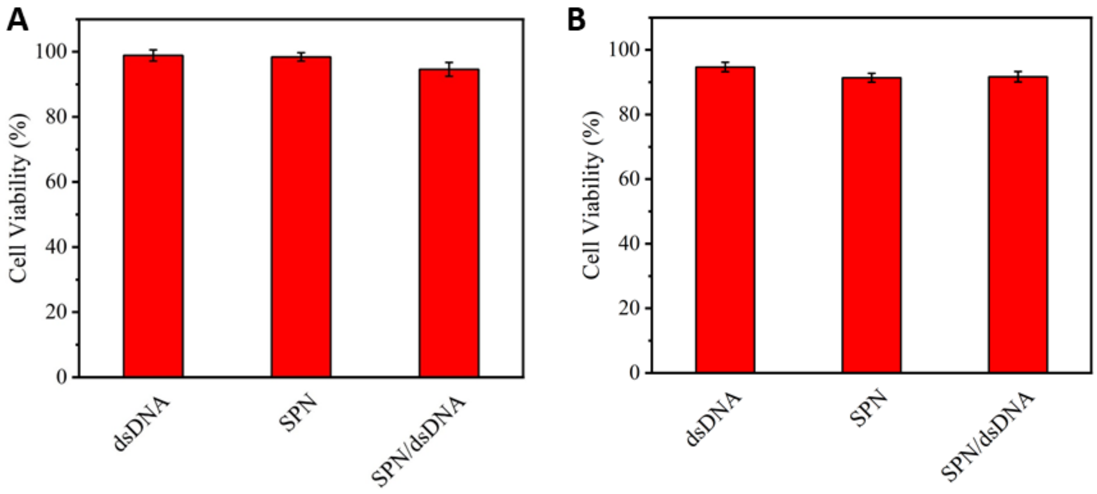

Figure S5. Cytotoxicity analysis of SPN/dsDNA system. MCF-7 cells (A) or L-O2 cells (B) were treated with indicated samples for 24 hours at dsDNA concentration of 100 nM. Cell viability was analyzed by CCK-8 assay. Data expressed as mean ± SD (n=3).
